# Artificial Intelligence for Risk–Benefit Assessment in Hepatopancreatobiliary Oncologic Surgery: A Systematic Review of Current Applications and Future Directions on Behalf of TROGSS—The Robotic Global Surgical Society

**DOI:** 10.3390/cancers17203292

**Published:** 2025-10-11

**Authors:** Aman Goyal, Michail Koutentakis, Jason Park, Christian A. Macias, Isaac Ballard, Shen Hong Law, Abhirami Babu, Ehlena Chien Ai Lau, Mathew Mendoza, Susana V. J. Acosta, Adel Abou-Mrad, Luigi Marano, Rodolfo J. Oviedo

**Affiliations:** 1 Department of General Surgery, Mahatma Gandhi Medical College and Research Institute, Sri Balaji Vidhyapeeth, Pondicherry-Cuddalore Rd., ECR, Pillayarkuppam 607402, Puducherry, India; amangoyal@student.sbvu.ac.in; 2 Adesh Institute of Medical Sciences and Research, Bathinda 151109, Punjab, India; 3 Department of Experimental and Clinical Pharmacology, Medical University of Warsaw, 02-927 Warsaw, Poland; s080772@student.wum.edu.pl; 4 Department of Surgery, Sam Houston State University College of Osteopathic Medicine, Conroe, TX 77304, USA; jjp064@shsu.edu (J.P.); itb004@shsu.edu (I.B.); 5 Department of Health and Science, Hillsborough College, Tampa, FL 33614, USA; cmacias3@hawkmail.hccfl.edu; 6 Department of Internal Medicine, Hospital Seri Manjung, Perak 32040, Malaysia; shenghonglaw9118@gmail.com; 7 School of Medicine, Our Lady of Fatima University, Manila 1440, Philippines; ababu0477val@student.fatima.edu.ph; 8 Faculty of Medicine, Manipal University College Malaysia, Melaka 75150, Malaysia; 231006021@scholar.manipal.edu.my; 9 Department of Surgery, University of Houston Tilman J. Fertitta Family College of Medicine, Houston, TX 77021, USA; mmendo31@cougarnet.uh.edu; 10 Universidad Central de Venezuela, Caracas 1051, Venezuela; susanavictoriaacosta@gmail.com; 11 Department of Surgery, Centre Hospitalier Universitaire d’Orléans, 45100 Orléans, France; adel.abou-mrad@orange.fr; 12 Department of Medicine, Academy of Applied Medical and Social Sciences-AMiSNS, Akademia Medycznych I Spolecznych Nauk Stosowanych, 82-300 Elbląg, Poland; l.marano@amisns.edu.pl; 13 Department of General Surgery and Surgical Oncology, “Saint Wojciech” Hospital, “Nicolaus Copernicus” Health Center, 80-462 Gdańsk, Poland; 14 Department of Surgery, Nacogdoches Medical Center, Nacogdoches, TX 75965, USA

**Keywords:** artificial intelligence, HPB surgery, risk assessment, machine learning, oncologic surgery, decision-making, postoperative complications, survival prediction

## Abstract

**Background:** Hepatopancreatobiliary (HPB) surgery is among the most complex domains in oncologic care, where decisions entail significant risk–benefit considerations. Artificial intelligence (AI) has emerged as a promising tool for improving individualized decision-making through enhanced risk stratification, complication prediction, and survival modeling. However, its role in HPB oncologic surgery has not been comprehensively assessed. **Methods:** This systematic review was conducted in accordance with PRISMA guidelines and registered with PROSPERO ID: CRD420251114173. A comprehensive search across six databases was performed through 30 May 2025. Eligible studies evaluated AI applications in risk–benefit assessment in HPB cancer surgery. Inclusion criteria encompassed peer-reviewed, English-language studies involving human s ubjects. Two independent reviewers conducted study selection, data extraction, and quality appraisal. **Results:** Thirteen studies published between 2020 and 2024 met the inclusion criteria. Most studies employed retrospective designs with sample sizes ranging from small institutional cohorts to large national databases. AI models were developed for cancer risk prediction (n = 9), postoperative complication modeling (n = 4), and survival prediction (n = 3). Common algorithms included Random Forest, XGBoost, Decision Trees, Artificial Neural Networks, and Transformer-based models. While internal performance metrics were generally favorable, external validation was reported in only five studies, and calibration metrics were often lacking. Integration into clinical workflows was described in just two studies. No study addressed cost-effectiveness or patient perspectives. Overall risk of bias was moderate to high, primarily due to retrospective designs and incomplete reporting. **Conclusions:** AI demonstrates early promise in augmenting risk–benefit assessment for HPB oncologic surgery, particularly in predictive modeling. However, its clinical utility remains limited by methodological weaknesses and a lack of real-world integration. Future research should focus on prospective, multicenter validation, standardized reporting, clinical implementation, cost-effectiveness analysis, and the incorporation of patient-centered outcomes.

## Simple Summary

Hepatopancreatobiliary (HPB) cancer surgery is one of the most challenging areas in cancer treatment, requiring highly accurate decisions about risks and benefits for each patient. In recent years, artificial intelligence (AI) has shown promise in helping doctors predict important outcomes, such as the likelihood of cancer, possible complications after surgery, and long-term survival. We reviewed all available studies that used AI to assist in these decisions for HPB cancer surgery. We found that while AI can make accurate predictions in research settings, most studies were small, retrospective, and rarely tested in real-world clinical practice. Important factors such as cost, patient perspectives, and integration into everyday surgical care have not yet been addressed. This review highlights the potential of AI in improving decision-making for complex cancer surgeries and outlines the next steps needed to bring these tools into routine clinical use.

## 1. Introduction

Hepatopancreatobiliary (HPB) surgery represents one of the most technically demanding and high-risk domains within the field of oncologic and complex abdominal surgery. It encompasses major liver resections, pancreaticoduodenectomy (Whipple procedures), and bile duct reconstructions, often performed for malignancy or severe benign disease [1]. Patients undergoing HPB surgery frequently identified with advanced-stage disease, substantial comorbidities, and a diminished physiological reserve [2]. Consequently, perioperative decision-making carries considerable stakes with significant potential for both benefit and harm. In this context, accurate and individualized risk/benefit assessment is essential for guiding appropriate patient selection, preoperative optimization, and surgical planning.

Historically, risk stratification in HPB surgery has relied on clinical scoring systems and surgeon experience. Certain classifications, such as the American Society of Anesthesiologists (ASA) classification, the Charlson Comorbidity Index, and procedure-specific morbidity calculators, have provided some degree of risk estimation [3,4]. However, such models often lack generalizability, as they often fail to capture the heterogeneity of patient populations or the evolving complexity of contemporary surgical care. In addition, traditional risk estimates offer limited value when applied to individual, patient-centered clinical decisions.

In recent years, artificial intelligence (AI) has shown growing potential to transform surgical decision-making [5]. Machine learning (ML) methods, in particular, can integrate and analyze large volumes of clinical, imaging, and molecular data, enabling risk assessments that move beyond the limitations of traditional tools [6]. Unlike conventional models, which rely on linear associations and a limited set of predefined variables, AI-based approaches can identify complex, nonlinear patterns within diverse datasets. This allows for dynamic, patient-specific predictions that better reflect the variability encountered in clinical practice. Such precision is particularly relevant in HPB surgery, where subtle differences in patient and tumor characteristics may have a significant impact on outcomes.

Thus far, several proof-of-concept studies suggest that AI-based models may outperform conventional risk scores in predicting key outcomes, such as perioperative complications, mortality, length of stay, and long-term survival in HPB surgery. AI applications have also been explored across various phases of care, including preoperative imaging, intraoperative guidance, and postoperative monitoring, highlighting their potential throughout the surgical pathway. However, integration into routine clinical practice remains limited. Many models rely on retrospective, single-center data with limited external validation, raising concerns about their generalizability. Questions also persist regarding interpretability, usability in real-world settings, and the broader ethical implications of algorithm-guided decision-making.

AI’s potential to support shared decision-making is compelling. This process requires an individualized balance between medical evidence and individual patient values. Thoughtfully designed AI-powered models can help clarify complex risks and benefits using extensive patient data to provide tailored predictions. This promotes transparent discussions, better-informed consent, and, ultimately, care plans that are more aligned with patient goals.

Still, it is critical to recognize that AI cannot replace surgical judgment or human empathy. Its true value lies in complementing clinical expertise, enhancing rather than taking over the surgeon’s role. To realize this potential, a thorough evaluation of AI applications in HPB surgery is essential, encompassing technical performance, clinical integration, and patient-centered outcomes. A systematic review is therefore timely, providing an opportunity to critically assess existing work, identify gaps, and set a direction for future research and innovation in this evolving field.

## 2. Materials and Methods

### 2.1. Search Strategy

A comprehensive literature search was conducted across five electronic databases: PubMed, Embase, Scopus, Web of Science, and the Cochrane Library. The search aimed to identify studies on AI’s impact on risk–benefit assessment and clinical decision-making in HPB oncologic surgery. The search strategy combined Medical Subject Headings (MeSHs) with relevant free-text keywords related to AI applications in oncologic surgery. The review was conducted in accordance with the Preferred Reporting Items for Systematic Reviews and Meta-Analyses Protocols (PRISMA-P 2020) guidelines [7]. We registered the protocol for this systematic review in the International Prospective Register of Systematic Reviews (PROSPERO) under registration number CRD420251114173. Search terms and subject headings included variations of terms related to HPB malignancies (e.g., “pancreatic cancer,” “hepatobiliary neoplasms”), AI methodologies (e.g., “machine learning,” “computational intelligence,” “computer vision”), and risk–benefit concepts (e.g., “risk assessment,” “benefit–risk analysis,” “clinical decision-making”). The full search strategy for each database is available in the Supplementary Materials (Table S1). Searches covered database inception to 30 May 2025, without geographic restrictions. Duplicate records were identified and removed using Rayyan software (https://www.rayyan.ai/ accessed on 30 May 2025) [8].

### 2.2. Eligibility Criteria

Inclusion criteria for this manuscript were studies evaluating AI applications specifically designed to assess risk–benefit outcomes in HPB oncologic surgery. All peer reviewed articles conducted on human subjects were eligible, with no language or publication date restriction. Excluded studies consisted of narrative reviews, editorials, commentaries, opinion pieces, studies not focused on AI-driven risk–benefit calculations, or purely theoretical papers without clinical validation. Additionally, studies involving animals or those that did not involve human subjects were also excluded.

### 2.3. Data Extraction

Four reviewers independently screened titles and abstracts using Rayyan software [8]. Full texts of eligible studies were reviewed, and any disagreements regarding inclusion were resolved by consensus. Data extraction was performed by six reviewers in total, the initial four from the screening process plus two additional reviewers. Extracted data were compiled into a master table and cross-checked for accuracy. Across studies, data inputs included structured clinical variables (e.g., age, comorbidities, labs), imaging (e.g., EUS, CT, digital pathology), and registry-level demographics and diagnostic codes. The following variables were collected from each included study:

- Study characteristics: titles, authors, year of publication, study design, and country of origin.
- Population characteristics: sample size (total number of participants), surgical population.
- AI technologies: data source, AI model types, best-performing model, prediction target.
- Outcomes: effect sizes (OR, RR, HR) and corresponding 95% confidence intervals (CI) for AUROC validation, sensitivity, specificity, key predictors, external validation, and clinical use.

### 2.4. Quality Assessment and Synthesis Technique

The methodological quality of the included studies was assessed using the Newcastle–Ottawa Scale (NOS) [9] for cohort studies and the QUADAS-2 tool [10] for studies evaluating diagnostic or predictive accuracy. Quality assessment was conducted by multiple reviewers, with discrepancies addressed collaboratively. Given the anticipated heterogeneity in study designs, AI models, and outcome measures, the primary data synthesis was narrative. Key findings were summarized in structured tables. Results are presented descriptively with tables and figures provided to support transparency.

## 3. Results

### 3.1. Study Selection

**Figure 1:**
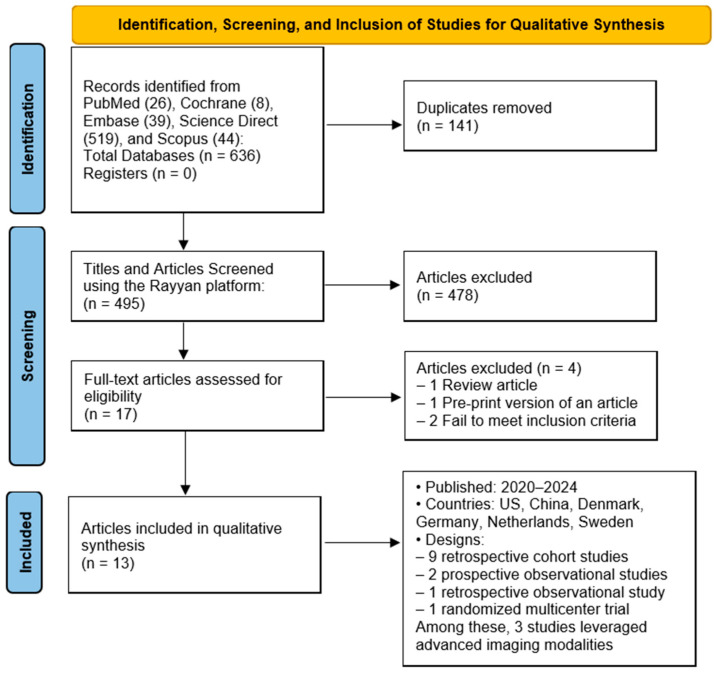
PRISMA flow diagram illustrating the screening, inclusion, and exclusion processes for studies incorporated in this work.

Based on a systematic search conducted across five databases, PubMed (26), Cochrane (8), Embase (39), Science Direct (519), and Scopus (44), a total of 636 articles were retrieved. After the removal of 141 duplicates, 495 titles and abstracts were screened using the Rayyan platform. Of these, 478 studies were excluded after title and abstract screening due to irrelevance or non-compliance with inclusion criteria. A full-text review was performed for the remaining 17 articles. Following this step, four articles were excluded for the following reasons: one was a review article, one was a pre-print version of an article included in these 17 articles, and two did not fulfill the inclusion criteria. Finally, 13 studies met the inclusion criteria for qualitative synthesis. The included studies were published between 2020 and 2024. The study selection process is illustrated in Figure 1, created using the web application developed by Haddaway et al. [11].

### 3.2. Study Characteristics

The included studies originated primarily from the United States 5 (38%), with other studies from China 3 (23%), Denmark 2 (15%), Germany 1 (7.6%), the Netherlands 1 (7.6%), and Sweden 1 (7.6%). These studies employed a range of methodological designs, including nine retrospective cohort studies, two prospective observational studies (including one post hoc analysis), one retrospective observational study, and one randomized multicenter trial. Among these, three studies, those by Leupold et al. [12], Màlyi et al. [13], and Machicado et al. [14], specifically leveraged advanced imaging modalities (e.g., EUS-nCLE, digital pathology, or confocal laser endomicroscopy) as primary data sources. Table 1 illustrates the characteristics of all included studies.

### 3.3. Study Objectives and Data Sources

The primary objectives of these studies centered on the application of AI in oncologic surgery, specifically for cancer risk prediction (n = 9), postoperative complication modeling (n = 4), and long-term prognostication (n = 3). The data sources varied, including national cancer registries (e.g., SEER, DNPR), institutional electronic health records (EHRs), imaging databases, and multi-institutional datasets (e.g., ACS NSQIP, TriNetX). Sample sizes varied substantially across studies, from small imaging-based cohorts of 64 patients to expansive national registries encompassing over four million individuals.

### 3.4. Artificial Intelligence (AI) Models Applied

A broad spectrum of ML and deep learning (DL) techniques were used across the studies. Commonly applied models included Random Forest, XGBoost, Cox regression, Decision Trees, Gradient Boosting Machines, Convolutional Neural Networks (CNNs), Transformers, and Artificial Neural Networks (ANNs). Some studies compared these AI models with traditional scoring systems, while others employed multimodal approaches that integrated clinical, imaging, and histopathological data to optimize model performance. Table 2 illustrates the AI models applied from the included studies.

### 3.5. Model Performance Across Clinical Use Cases

#### 3.5.1. Cancer Risk Prediction

Across studies focusing on pancreatic cancer risk prediction, AI models consistently demonstrated superior discriminatory performance compared to traditional scoring systems. For instance, Cichosz et al. (2024) [18] achieved an AUROC of 0.78 using a Random Forest classifier to distinguish pancreatic cancer–related diabetes from type 2 diabetes in a Danish national cohort. In the United States, Khan et al. (2024) [15] utilized an XGBoost model to distinguish the same pathologies in a large multi-institutional dataset and reported an AUROC of 0.80, outperforming established clinical tools such as END-PAC and the Boursi model. Placido et al. (2023) [21] developed a Transformer-based model for predicting pancreatic cancer from a wider range of diagnoses trained on over 6 million patients. The latter study achieved an AUROC of 0.879 in Danish data and 0.710 in external validation using a U.S. Veterans Affairs cohort, demonstrating both strong predictive potential and notable limitations in generalizability across populations. Table 3 demonstrates the AUROC scores regarding cancer risk prediction evaluation in the included studies.

#### 3.5.2. Postoperative Complication Prediction

Four studies, by Wang H. et al. (2024) [16], Màlyi A. et al. (2024) [13], Ingwersen E.W. et al. (2023) [20], and Merath K. et al. (2020) [24], focused on modeling the risk of postoperative complications, particularly clinically relevant postoperative pancreatic fistula (CR-POPF) and delayed gastric emptying (DGE). Wang H. et al. (2024) [16] reported an AUROC of an internal test set in China of 0.88 for survival and 0.79 for complications with a sensitivity of 67% for survival rate and 77% for complications. Specificity was not reported. Màlyi A. et al. (2024) [13], with a model based on the quantification of fibrotic tissue content, reported an internal AUROC of 0.73 and a sensitivity and specificity of 80% and 62%, respectively, using a generalized linear model. In the Netherlands, Ingwersen E.W. et al. (2023) [20] used gradient boosting methods and achieved AUROCs of approximately 0.74 for CR-POPF, marginally outperforming logistic regression. However, models predicting DGE generally showed lower discrimination, with AUROCs around 0.59. Sensitivity and specificity were not reported. Finally, Merath K. et al. (2020) [24] reported an AUROC of 0.74 for overall complications. Sensitivity and specificity were not reported.

Despite these encouraging results, none of the complication models reported external validation, and calibration statistics were generally lacking, limiting confidence in their real-world applicability. Table 4 illustrates the postoperative complication prediction across the included studies.

#### 3.5.3. Survival Prognostication

Two studies, by Wang H. et al. (2024) [16] and Aronsson L. et al. (2021) [23], examined the use of AI models to predict long-term oncologic outcomes, such as five-year disease-specific survival. Wang H. et al. (2024) [16] applied Decision Tree models to predict both postoperative complications and 1-year survival after pancreatoduodenectomy, reporting an AUROC of 0.88 for survival prediction with a sensitivity of 67%. Specificity was not reported. Aronsson L. et al. (2021) [23] used Artificial Neural Networks to predict 5-year survival for invasive IPMN, reporting an F1 score of 0.89 and an accuracy of 0.82. The sensitivity and specificity reports were 95% and 42%, respectively. Despite these promising results, most prognostic models lacked external validation and did not report calibration or decision curve analyses. Thus, while internal performance appeared to be robust, generalizability and clinical interpretability remain uncertain. Table 5 illustrates the survival prognostication across the included studies.

### 3.6. Validation and Clinical Integration

Five studies, by Khan S. et al. (2024) [15], Hu K. et al. (2024) [17], Cichosz SL. et al. (2024) [18], Chen W. et al. (2023) [19], and Placido D. et al. (2023) [21], performed some form of external validation by applying their models to independent cohorts or split datasets from separate institutions. Among these, Chen W. et al. (2023) [19] externally validated their machine learning model on a U.S. Veterans Affairs cohort, while Placido D. et al. (2023) [21] tested a Transformer model trained on Danish health data against a U.S. Veterans Affairs population, demonstrating performance degradation across populations. Khan S. et al. (2024) [15] and Cichosz SL. et al. (2024) [18] used large registry-based cohorts with hold-out test sets or pseudo-external validation splits within national data. Hu K. et al. (2024) [17] employed separate multicenter hospital datasets for validation of a deep learning pathology model for PDAC survival prediction. Notably, only Chen W. et al. (2023) [19] and Placido et al. (2023) [21] demonstrated preliminary integration of AI tools into prototype electronic health record workflows, enabling real-time or near-real-time risk stratification. However, detailed decision curve analysis and calibration reporting were rarely performed, limiting the interpretability and the practical applicability of these AI models in real-world clinical settings. Table 6 illustrates the validation and clinical integration across the included studies.

Because the included studies were heterogeneous and unsuitable for meta-analysis, formal assessments of study bias, heterogeneity, and reporting bias could not be performed in the Results and are instead discussed narratively in Section 4.2.

## 4. Discussion

This systematic review evaluated the role of AI in risk–benefit assessment and clinical decision-making within HPB oncologic surgery. Although AI has been explored extensively across other surgical specialties, its application in the HPB context remains comparatively limited. Our findings show that AI models have been employed most commonly for cancer risk prediction, postoperative complication modeling, and long-term survival prognostication. In these areas, AI systems generally demonstrated strong internal performance and, in many cases, outperformed traditional statistical approaches. Across studies, AI models typically achieved AUROCs in the 0.74–0.88 range, whereas traditional logistic regression or clinical scores such as END-PAC and Boursi achieved lower values (0.63–0.71), underlining incremental but not yet definitive clinical value. However, the clinical translation of these models remains modest, and the quality and consistency of reporting across studies varied considerably.

From the perspective of patients and an economic impact viewpoint, a notable gap in the current literature is the absence of studies investigating patient perspectives on the integration of AI into surgical decision-making [25]. As these technologies are introduced into clinical environments, understanding how patients perceive algorithm-supported care will be critical for creating trust, promoting transparency, and supporting shared decision-making. Without this insight, there is a risk that AI implementation may proceed in ways that overlook individual preferences, values, or concerns, which could compromise patient engagement or even undermine the therapeutic alliance between patients and clinicians.

Similarly, none of the included studies evaluated the cost-effectiveness or broader economic implications of deploying AI systems in surgical workflows [26]. Although many AI models show promising accuracy in retrospective datasets, their development, validation, and maintenance often require substantial financial and technical investment. Without rigorous economic evaluation, it is difficult to determine whether these tools offer practical value beyond their performance metrics. Future research should incorporate patient-centered outcomes and health economic analyses to determine whether AI offers added value not only in terms of predictive accuracy but also in cost-efficiency and patient satisfaction. Addressing these economic dimensions would help establish a more complete framework for evaluating AI in surgical practice, informing not only clinical decision-making but also strategic planning and policy development at institutional and national levels.

In parallel, the absence of patient-centered and ethical considerations across the reviewed studies represents an important gap. As AI becomes more present in surgical planning, understanding how patients interpret algorithm-based recommendations is essential to support shared decision-making. A lack of attention to concerns such as transparency, data use, and equitable access may affect how these technologies are received in clinical settings. Ethical issues, particularly those related to consent, privacy, and fairness, also remain underexplored. Moving forward, studies should aim to include qualitative and ethics-oriented perspectives to ensure that AI tools reflect not only technical performance, but also the values and expectations of the individuals they are designed to serve.

### 4.1. Observations in Producing an Effective Model

Several of the included studies made observations about factors that contributed to the effectiveness of their AI models. Placedo et al. noted that AI predicted pancreatic cancer when given the time-onset of predictive factors rather than just the presence of predictive factors. This temporal context allowed the model to identify patterns of disease emergence more realistically and aligned with how clinical symptoms and risk factors often evolve. This group also noted that their model’s accuracy softened when applied to a region outside of what the model was trained on, suggesting the need to train models in each new area to maintain generalizability. This reinforces a broader challenge in AI deployment, which is the trade-off between model performance and portability, particularly in geographically or demographically distinct populations.

One group supports the use of multiple models together to improve efficiency [19], while another group provides that complexity can be reduced by selecting variables to eliminate from the machine’s training without compromising efficacy [23]. These observations indicate an important balancing act between model accuracy and interpretability, especially when considering clinical adoption. For models that read histologic slides, simple global image processing may be sufficient compared to models that were fed histologic decomposition data, as they performed equivocally [17]. This finding raises questions about the value of more computationally expensive preprocessing steps and suggests that, in some contexts, streamlined approaches may be sufficient.

Wang et al. reported strong results using their decision-tree-based model on a 9:1 training to testing ratio. This underscores how model performance can be sensitive to the training strategy employed, particularly the partitioning of data for internal validation. The decision tree is highlighted by another group as a modality that yields accurate results and remains relatively easy to interpret [24]. This interpretability is a critical advantage in clinical settings where transparency and trust in algorithmic outputs are important. Lastly, Ingwersen et al. noted that machine learning shows particular usefulness over linear regression when unstructured data is included in the comparison, such as that from MRI and CT scans. This shows AI’s capacity to handle complex and high-dimensional data types that traditional statistical approaches may not accommodate well, which is especially relevant in the imaging-rich environment of HPB surgery.

### 4.2. Limitations and Risk of Bias

This systematic review has several limitations. Despite a comprehensive search across multiple databases, it is possible that relevant studies in the gray literature or unpublished sources were not captured. This may have led to an incomplete representation of the evidence base, particularly given the rapid and often fragmented nature of research dissemination in the field of AI. Additionally, the included studies displayed considerable heterogeneity in terms of study design, patient populations, cancer subtypes, AI model architecture, and outcome measures. This methodological diversity limited our ability to conduct meaningful quantitative synthesis and restricted the generalizability of aggregated findings.

Another key limitation relates to the inconsistent quality and transparency of reporting across studies. Few studies adhered to established reporting guidelines that are necessary to assess clinical applicability, including the documentation of model calibration, external validation, strategies for handling missing data, and the use of decision curve analysis. The absence of these critical elements reduces interpretability and weakens the strength of evidence supporting real-world deployment of the proposed models.

A further limitation relates to the considerable heterogeneity in sample size and data source across the included studies. While some models were developed using highly granular datasets from single centers with fewer than 100 patients, others relied on national or multinational registries encompassing millions of records. This variability complicates direct comparison between studies and may affect both the internal consistency and the external validity of the models. For instance, the Transformer-based model developed by Placido et al. achieved a high AUROC of 0.879 when validated within the Danish National Patient Registry but demonstrated reduced performance (AUROC 0.710) when applied to an external cohort from the U.S. Veterans Affairs system. This decline underscores the risks of geographic and population-specific overfitting, particularly when training data lacks demographic or clinical diversity [27]. Moreover, many studies that relied on large national or multinational datasets offered limited reporting on key aspects such as data completeness, the handling of missing values, and underlying biases within the source populations. These omissions raise concerns about the transparency and reproducibility of findings, especially when registry data may include heterogeneous coding practices or an imbalanced representation of clinical subgroups. Without adequate detail on data preprocessing and quality control, it becomes challenging to assess the reliability of model outputs or compare performance across studies.

Although several AI models demonstrated promising results, limited reporting of performance metrics and a lack of transparency in model architecture hindered a thorough assessment of interpretability. As clinician trust in AI depends strongly on explainability, particularly for complex models such as neural networks and Transformers, future research should prioritize incorporating interpretability frameworks and transparent reporting standards to support clinical adoption.

The risk of bias was judged to be moderate to high in many studies, often stemming from a retrospective design, an absence of predefined thresholds, and a lack of blinding in outcome assessment. Most studies relied on retrospective data, which may be subject to selection bias, incomplete records, and unmeasured confounders. Furthermore, the observational nature of retrospective designs restricts the ability to draw causal conclusions from associations identified by AI models. Without prospective validation or randomization, it remains unclear whether these algorithms are capturing true mechanistic relationships or merely reflecting correlations within the data.

The infrequent use of prospective methodologies and external datasets further constrains the robustness and reproducibility of reported results. In addition, the potential for publication bias should be acknowledged. As is common in emerging areas of research, studies with favorable or novel findings may be disproportionately represented in the published literature, skewing the perceived effectiveness of AI applications in HPB oncologic surgery.

### 4.3. Future Directions

Further research is needed to move beyond the development of AI models and toward their practical, ethical, and effective integration into clinical care. Prospective, multicenter studies that include diverse patient populations are critical to improving the generalizability and clinical relevance of AI in HPB oncologic surgery. These designs would allow for a more accurate assessment of how AI performs under real-world conditions, across various healthcare systems and demographic groups.

Equally important is the emphasis on external validation, consistent reporting, and the creation of tools that are both technically sound and clinically usable. User-friendly design, interpretability, and seamless integration into existing clinical workflows should be prioritized to promote uptake by surgical teams. The use of standardized reporting frameworks, including TRIPOD-ML, PROBAST-AI, and CONSORT-AI, will help ensure methodological transparency and comparability across future studies.

Cross-disciplinary collaboration between clinicians, data scientists, implementation researchers, and healthcare administrators will be essential to ensure that these models are not only accurate but also ethically designed, interpretable, and aligned with the realities of surgical practice. Beyond technical validation, future studies should also explore patient perspectives, assess the cost-effectiveness of AI implementation, and evaluate the long-term impact of these systems on health outcomes and care delivery. Together, these efforts will support the meaningful integration of AI into HPB surgery, enabling more informed decisions, fostering patient trust, and ultimately improving clinical outcomes.

## 5. Conclusions

This systematic review emphasized the growing convergence between technological advancements and complex surgical procedures, placing AI as a potentially transformative tool to optimize risk–benefit analysis in HPB oncologic surgery. Through multiple methodologies and applications to the clinic, AI is reshaping the risk analysis and the prediction of HPB oncology outcomes. While the progress in model development has been rapidly evolving, the translation into a meaningful clinical integration is still ongoing.

AI’s true potential not only lies in its precision but in its ability to support clinical judgment when applied deliberately, requiring rigorous validation, multidisciplinary collaboration, and alignment with real-world surgical decision-making to realize its transformative potential in HPB oncology. While current AI models reflect improved predictive performance in cancer risk stratification, complication predictions, and survival analysis compared to the traditional methodologies, use in clinical practice still remains limited with heterogeneous factors such as limited external validation, heterogeneous reporting, and a lack of prospective, multicenter studies.

Notably, none of the included studies assessed patient perspectives or cost-effectiveness, which are critical dimensions for real-world adoption. These omissions urge a paradigm shift from model-centric algorithmic refinement to ethically grounded, patient-focused, and clinically embedded AI solutions. Bridging this gap can be attained through collaborative efforts among clinicians, data scientists, and policymakers to prioritize real-world applicability, transparency, and equitable implementation in HPB surgical care. Such integration will allow AI to become a valuable ally in HPB oncology, empowering clinicians to navigate high-stakes decisions while improving outcomes for patients facing complex oncologic operations by guiding safer, more individualized approaches.

## Figures and Tables

**Table 1:** Characteristics of all included manuscripts (n = 13) based on authors, journal, publication year, country, study type, and sample size.

Authors	Journal	Publication Year	Country	Study Type	Sample Size
Leupold M, et al. [12]	*Gastrointestinal Endoscopy*	2024	USA	Observational	64
Khan S, et al. [15]	*Journal of Clinical Gastroenterology*	2024	USA	Retrospective Cohort	81,213
Wang H, et al. [16]	*Frontiers in Oncology*	2024	China	Retrospective Cohort	749
Hu K, et al. [17]	*BMC (BioMed Central) Gastroenterology*	2024	China	Retrospective Cohort	142
Màlyi A, et al. [13]	*HPB (Hepato-Pancreato-Biliary)*	2024	Germany	Randomized Multicenter Trial	320
Cichosz SL, et al. [18]	*Computer Methods and Programs in Biomedicine*	2024	Denmark	Retrospective Cohort	1432
Chen W, et al. [19]	*Pancreatology*	2023	USA	Retrospective Cohort	4,500,000
Ingwersen EW, et al. [20]	*Surgery*	2023	Netherlands	Retrospective Observational	4912
Placido D, et al. [21]	*Nature Medicine*	2023	Denmark	Retrospective Cohort	9,200,000
Li Q, et al. [22]	*Disease Markers*	2022	China	Retrospective Cohort	47,919
Machicado JD, et al. [14]	*Gastrointestinal Endoscopy*	2021	USA	Prospective Single-Center	35
Aronsson L, et al. [23]	*PLOS One*	2021	Sweden	Retrospective Cohort	440
Merath K, et al. [24]	*Journal of Gastroenterology Surgery*	2020	USA	Retrospective Cohort	15,657

**Table 2:** Artificial intelligence (AI) models applied in the included manuscripts.

Authors	Publication Year	Country	Sample Size	AI Model Used	Best Model *
Leupold M, et al. [12]	2024	USA	64	EUS-nCLE	Combined model (EUS-nCLE + 2024 Kyoto High-Risk Stigmata)
Khan S, et al. [15]	2024	USA	81,213	XGBoost, END-PAC Boursi	XGBoost
Wang H, et al. [16]	2024	China	749	Decision Tree	Decision Tree
Hu K, et al. [17]	2024	China	142	DenseNet121, ResNet18 MobileNet_v3_small	Combined Model (Pathological Risk Signature + Clinical Risk Signature)
Màlyi A, et al. [13]	2024	Germany	320	QuPath AI Generalized Linear Model (GLM)	Generalized Linear Model (GLM)
Cichosz SL, et al. [18]	2024	Denmark	1432	Random Forest (RF)	Random Forest (RF)
Chen W, et al. [19]	2023	USA	4,500,000	Random Survival Forest (RSF) eXtreme Gradient Boosting (XGB) Cox Proportional Hazards (COX)	eXtreme Gradient Boosting (XGB)
Ingwersen EW, et al. [20]	2023	Netherlands	4912	Random Forest Neural Network Support Vector Machine Gradient Boosting	Gradient Boosting
Placido D, et al. [21]	2023	Denmark	9,200,000	Bag-of-Words Multilayer Perceptron (MLP) Gated Recurrent Unit (GRU) Transformer	Transformer
Li Q, et al. [22]	2022	China	47,919	Random Forest (RF) XGBoost SVM Deep Neural Network (DNN) Logistic Regression (LR)	Random Forest (RF)
Machicado JD, et al. [14]	2021	USA	35	Segmentation-Based Model (SBM) Holistic-Based Model (HBM)	Holistic-Based Model (HBM)
Aronsson L, et al. [23]	2021	Sweden	440	Artificial Neural Network (ANN) LASSO Logistic Regression	Artificial Neural Networks (ANNs)
Merath K, et al. [24]	2020	USA	15,657	Decision Tree	Decision Tree

* Best model refers to the algorithm reported by the study authors as achieving the highest performance (AUROC/F1/accuracy).

**Table 3:** AUROC scores to assess cancer risk prediction in included manuscripts.

Authors	Publication Year	Country	Sample Size	AUROC (DK)	AUROC (US Cross-Validation)	AUROC (US Independent Training)	AUROC (Internal)
Cichosz SL, et al. [18]	2024	Denmark	1432	0.78 (CI 0.75–0.83)	N/A	N/A	N/A
Khan S, et al. [15]	2024	USA	81,213	N/A	0.81 (XGBoost); 0.66 (END-PAC); 0.71 (Boursi)	N/A	0.80 (XGBoost); 0.63 (END-PAC); 0.68 (Boursi)
Placido D, et al. [21]	2023	Denmark	9,200,000	0.879	0.710 (Danish-trained model)	0.775 (US-VA model)	N/A

**Table 4:** Postoperative Complication Prediction.

Authors	Publication Year	Country	Sample Size	AUROC (Internal)	Sensitivity	Specificity
Wang H, et al. [16]	2024	China	749	0.79	77%	N/A
Màlyi A, et al. [13]	2024	Germany	320	0.73	80%	62%
Ingwersen EW, et al. [20]	2023	Netherlands	4912	CR-POPF Gradient Boosting (ML model) = 0.74 DGE-Both models (ML and logistic regression) = 0.59	N/A	N/A
Merath K, et al. [24]	2020	USA	15,657	0.74	N/A	N/A

**Table 5:** Survival prognostication across investigated studies.

Authors	Publication Year	Country	Sample Size	AUROC (US Cross Validation)	AUROC (Internal)	Sensitivity	Specificity
Wang H, et al. [16]	2024	China	749	N/A	0.88	67%	N/A
Aronsson L, et al. [23]	2021	Sweden	440	ANN1 = 0.82 F1 0.89	N/A	95%	42%

**Table 6:** Validation and clinical integration.

Authors	Publication Year	Country	Sample Size	External Validation	Clinical Use Case
Khan S, et al. [15]	2024	USA	81,213	N/A	Stratify patients with new-onset diabetes to identify high PDAC risk for early imaging (e.g., MRI, EUS).
Hu K, et al. [17]	2024	China	142	N/A	Prognosis of PDAC based on integrated histopathological and clinical data.
Cichosz SL, et al. [18]	2024	Denmark	1432	N/A	Future implication, not yet in clinical use Stratify patients ≥50 years old with new-onset diabetes (NOD) into high- vs. low-risk for pancreatic cancer (PCRD).
Chen W, et al. [19]	2023	USA	4,500,000	Externally validated using an independent cohort from the U.S. Veterans Affairs (VA) Health System	Risk stratification for PDAC screening using EHR data.
Placido D, et al. [21]	2023	Denmark	9,200,000	Cross-application of Danish-trained model to US-VA data	Identifying high-risk patients (e.g., top 0.1%) for cost-effective surveillance and early detection of pancreatic cancer.

## Data Availability

The datasets generated and/or analyzed during this study are not publicly available but may be obtained from the corresponding author upon reasonable request.

## Supplementary Materials

The following supporting information can be downloaded at https://www.mdpi.com/article/10.3390/cancers17203292/s1. Table S1: Search terms for the meta-analysis in PubMed, Scopus, Web of Science, and Science Direct databases.
