# Supplementary material for: Artificial Intelligence for Risk–Benefit Assessment in Hepatopancreatobiliary Oncologic Surgery: A Systematic Review of Current Applications and Future Directions on Behalf of TROGSS—The Robotic Global Surgical Society
Source: Cancers (Basel). 2025 Oct 11;17(20):3292. doi: 10.3390/cancers17203292 (PMC12564804; doi:10.3390/cancers17203292)
Supplement: Supplementary file 1 [file cancers-17-03292-s001.zip › cancers-3829459-supplementary.pdf]

Table S1: Comprehensive database search strategy

Database	Search Strategy
PubMed (n=26)	("Pancreatic Neoplasms"[Mesh] OR "Neoplasm, Pancreatic"[Title/Abstract] OR "Pancreatic Neoplasm"[Title/Abstract] OR "Neoplasms, Pancreatic"[Title/Abstract] OR "Pancreas Neoplasms"[Title/Abstract] OR "Neoplasm, Pancreas"[Title/Abstract] OR "Neoplasms, Pancreas"[Title/Abstract] OR "Pancreas Neoplasm"[Title/Abstract] OR "Cancer of Pancreas"[Title/Abstract] OR "Pancreas Cancers"[Title/Abstract] OR "Cancer of the Pancreas"[Title/Abstract] OR "Pancreas Cancer"[Title/Abstract] OR "Cancer, Pancreas"[Title/Abstract] OR "Cancers, Pancreas"[Title/Abstract] OR "Pancreatic Cancer"[Title/Abstract] OR "Cancer, Pancreatic"[Title/Abstract] OR "Cancers, Pancreatic"[Title/Abstract] OR "Pancreatic Cancers"[Title/Abstract] OR "Pancreatic Carcinoma"[Title/Abstract] OR "Carcinoma, Pancreatic"[Title/Abstract] OR "Carcinomas, Pancreatic"[Title/Abstract] OR "Pancreatic Carcinomas"[Title/Abstract] OR "Pancreatic Acinar Carcinoma"[Title/Abstract] OR "Acinar Carcinoma, Pancreatic"[Title/Abstract] OR "Acinar Carcinomas, Pancreatic"[Title/Abstract] OR "Carcinoma, Pancreatic Acinar"[Title/Abstract] OR "Carcinomas, Pancreatic Acinar"[Title/Abstract] OR "Pancreatic Acinar Carcinomas"[Title/Abstract]) **AND** ("Artificial Intelligence"[Mesh] OR "Intelligence, Artificial"[Title/Abstract] OR "Computer Reasoning"[Title/Abstract] OR "Reasoning, Computer"[Title/Abstract] OR "AI"[Title/Abstract] OR "Machine Intelligence"[Title/Abstract] OR "Intelligence, Machine"[Title/Abstract] OR "Computational Intelligence"[Title/Abstract] OR "Intelligence, Computational"[Title/Abstract] OR "Computer Vision Systems"[Title/Abstract] OR "Computer Vision System"[Title/Abstract] OR "System, Computer Vision"[Title/Abstract] OR "Systems, Computer Vision"[Title/Abstract] OR "Vision System, Computer"[Title/Abstract] OR "Vision Systems, Computer"[Title/Abstract] OR "Knowledge Acquisition (Computer)"[Title/Abstract] OR "Acquisition, Knowledge (Computer)"[Title/Abstract] OR "Knowledge Representation (Computer)"[Title/Abstract] OR "Knowledge Representations (Computer)"[Title/Abstract] OR "Representation, Knowledge (Computer)"[Title/Abstract]) **AND** ("Risk Assessment"[Mesh] OR "Risk Assessments"[Title/Abstract] OR "Assessment, Risk"[Title/Abstract] OR "Health Risk Assessment"[Title/Abstract] OR "Assessment, Health Risk"[Title/Abstract] OR "Health Risk Assessments"[Title/Abstract] OR "Risk Assessment, Health"[Title/Abstract] OR "Benefit-Risk Assessment"[Title/Abstract] OR "Assessment, Benefit-Risk"[Title/Abstract] OR "Benefit Risk Assessment"[Title/Abstract] OR "Benefit-Risk Assessments"[Title/Abstract] OR "Risk-Benefit Assessment"[Title/Abstract] OR "Assessment, Risk-Benefit"[Title/Abstract] OR "Risk Benefit Assessment"[Title/Abstract] OR "Risk-Benefit Assessments"[Title/Abstract] OR "Risks and Benefits"[Title/Abstract] OR "Benefits and Risks"[Title/Abstract] OR "Risk Analysis"[Title/Abstract] OR "Analysis, Risk"[Title/Abstract] OR "Risk Analyses"[Title/Abstract])
Scopus (n=44)	(TITLE-ABS-KEY("Neoplasm, Pancreatic" OR "Pancreatic Neoplasm" OR "Neoplasms, Pancreatic" OR "Pancreas Neoplasms" OR "Neoplasm, Pancreas" OR "Neoplasms, Pancreas" OR "Pancreas Neoplasm" OR "Cancer of Pancreas" OR "Pancreas Cancers" OR "Cancer of the Pancreas" OR "Pancreas Cancer" OR "Cancer, Pancreas" OR "Cancers, Pancreas" OR "Pancreatic Cancer" OR "Cancer, Pancreatic" OR "Cancers, Pancreatic" OR "Pancreatic Cancers" OR "Pancreatic Carcinoma" OR "Carcinoma, Pancreatic" OR "Carcinomas, Pancreatic" OR "Pancreatic Carcinomas" OR "Pancreatic Acinar Carcinoma" OR "Acinar Carcinoma, Pancreatic" OR "Acinar Carcinomas, Pancreatic" OR "Carcinoma, Pancreatic Acinar" OR "Carcinomas, Pancreatic Acinar" OR "Pancreatic Acinar Carcinomas")) **AND** (TITLE-ABS-KEY("Artificial Intelligence" OR "Intelligence, Artificial" OR "Computer Reasoning"

	OR "Reasoning, Computer" OR "AI" OR "Machine Intelligence" OR "Intelligence, Machine" OR "Computational Intelligence" OR "Intelligence, Computational" OR "Computer Vision Systems" OR "Computer Vision System" OR "System, Computer Vision" OR "Systems, Computer Vision" OR "Vision System, Computer" OR "Vision Systems, Computer" OR "Knowledge Acquisition (Computer)" OR "Acquisition, Knowledge (Computer)" OR "Knowledge Representation (Computer)" OR "Knowledge Representations (Computer)" OR "Representation, Knowledge (Computer)")) **AND** (TITLE-ABS-KEY("Risk Assessment" OR "Risk Assessments" OR "Assessment, Risk" OR "Health Risk Assessment" OR "Assessment, Health Risk" OR "Health Risk Assessments" OR "Risk Assessment, Health" OR "Benefit-Risk Assessment" OR "Assessment, Benefit-Risk" OR "Benefit Risk Assessment" OR "Benefit-Risk Assessments" OR "Risk-Benefit Assessment" OR "Assessment, Risk-Benefit" OR "Risk Benefit Assessment" OR "Risk-Benefit Assessments" OR "Risks and Benefits" OR "Benefits and Risks" OR "Risk Analysis" OR "Analysis, Risk" OR "Risk Analyses"))
Embase (n=39)	('pancreatic neoplasm'/exp OR 'pancreatic neoplasm':ab,ti OR 'pancreas cancer':ab,ti OR 'pancreatic carcinoma':ab,ti OR 'acinar carcinoma':ab,ti) **AND** ('artificial intelligence'/exp OR 'artificial intelligence':ab,ti OR 'machine intelligence':ab,ti OR 'computational intelligence':ab,ti OR 'computer vision':ab,ti OR 'knowledge acquisition':ab,ti OR 'knowledge representation':ab,ti) **AND** ('risk assessment'/exp OR 'risk assessment':ab,ti OR 'benefit risk assessment':ab,ti OR 'risk benefit analysis':ab,ti OR 'risk analysis':ab,ti OR 'risk benefit':ab,ti)
Cochrane (n=8)	([mh "Pancreatic Neoplasms"] OR "pancreatic neoplasm":ti,ab OR "pancreatic cancer":ti,ab OR "pancreatic carcinoma":ti,ab OR "acinar carcinoma":ti,ab) **AND** ([mh "Artificial Intelligence"] OR "artificial intelligence":ti,ab OR "machine intelligence":ti,ab OR "computational intelligence":ti,ab OR "computer vision":ti,ab OR "knowledge acquisition":ti,ab OR "knowledge representation":ti,ab) **AND** ([mh "Risk Assessment"] OR "risk assessment":ti,ab OR "benefit risk assessment":ti,ab OR"risk benefit":ti,ab OR "risk analysis":ti,ab)
Science Direct (n=519)	("pancreatic cancer" OR "pancreatic neoplasm" OR "pancreatic carcinoma")<br>AND<br>("artificial intelligence" OR "machine learning" OR "computer vision")<br>AND<br>("risk assessment" OR "risk-benefit" OR "benefit-risk analysis")

Late date of Search = 27th May, 2025

"Pancreatic Neoplasms"[Mesh]

Neoplasm, Pancreatic
Pancreatic Neoplasm
Neoplasms, Pancreatic
Pancreas Neoplasms
Neoplasm, Pancreas
Neoplasms, Pancreas
Pancreas Neoplasm
Cancer of Pancreas
Pancreas Cancers
Cancer of the Pancreas
Pancreas Cancer
Cancer, Pancreas
Cancers, Pancreas
Pancreatic Cancer
Cancer, Pancreatic
Cancers, Pancreatic
Pancreatic Cancers
Pancreatic Carcinoma
Carcinoma, Pancreatic
Carcinomas, Pancreatic
Pancreatic Carcinomas
Pancreatic Acinar Carcinoma
Acinar Carcinoma, Pancreatic
Acinar Carcinomas, Pancreatic
Carcinoma, Pancreatic Acinar
Carcinomas, Pancreatic Acinar
Pancreatic Acinar Carcinomas

"Artificial Intelligence"[Mesh]
Intelligence, Artificial
Computer Reasoning
Reasoning, Computer
AI (Artificial Intelligence)
Machine Intelligence
Intelligence, Machine
Computational Intelligence
Intelligence, Computational
Computer Vision Systems
Computer Vision System
System, Computer Vision
Systems, Computer Vision
Vision System, Computer
Vision Systems, Computer
Knowledge Acquisition (Computer)
Acquisition, Knowledge (Computer)
Knowledge Representation (Computer)
Knowledge Representations (Computer)
Representation, Knowledge (Computer)

"Risk Assessment"[Mesh]
Risk Assessments
Assessment, Risk

Health Risk Assessment
Assessment, Health Risk
Health Risk Assessments
Risk Assessment, Health
Benefit-Risk Assessment
Assessment, Benefit-Risk
Benefit Risk Assessment
Benefit-Risk Assessments
Risk-Benefit Assessment
Assessment, Risk-Benefit
Risk Benefit Assessment
Risk-Benefit Assessments
Risks and Benefits
Benefits and Risks
Risk Analysis
Analysis, Risk
Risk Analyses
